# Single-Nucleus Transcriptomic Analysis Reveals Important Cell Cross-Talk in Diabetic Kidney Disease

**DOI:** 10.3389/fmed.2021.657956

**Published:** 2021-04-21

**Authors:** Yi Wei, Xiang Gao, Aihua Li, Mengjun Liang, Zongpei Jiang

**Affiliations:** ^1^Department of Nephrology, The Sixth Affiliated Hospital, Sun Yat-sen University, Guangzhou, China; ^2^Department of Gastroenterology, The Sixth Affiliated Hospital, Sun Yat-sen University, Guangzhou, China

**Keywords:** single-cell sequencing, cell cross-talk, diabetic kidney disease, CellPhoneDB, glomerulotubular communication

## Abstract

Diabetic kidney disease (DKD) leads to the loss of renal function and cell cross-talk is one of the crucial mechanisms participating in the pathogenesis of DKD. However, the mechanisms of cell communication were not fully elucidated in previous studies. In this study, we performed cell cross-talk analysis using CellPhoneDB based on a single-nucleus transcriptomic dataset (GSE131882) and revealed the associations between cell communication-related genes and renal function, providing overall insight into cell communication in DKD. In addition, this study may facilitate the discovery of novel mechanisms, promising biomarkers, and therapeutic targets that are clinically beneficial to patients.

## Introduction

Diabetic kidney disease (DKD) (1, 2) is one of the most important microvascular complications of diabetic mellitus and a leading cause of renal function loss and end-stage renal disease (ESRD). Nevertheless, the mechanism of DKD is complex and not fully elucidated.

Renal parenchymal cells, resident immune cells, and infiltrating immune cells orchestrate active cell-to-cell interactions, thereby contributing to the development of DKD. Previous studies (3, 4) have revealed the significance of cell communication in the pathogenesis of DKD. Dmike et al. (5) deciphered the tubulovascular cross-talk mediated by vascular endothelial growth factor A. Wu et al. (6) found that high glucose-induced glomerular endothelial cell-derived exosomes trigger the epithelial-mesenchymal transition and podocytes dysfunctions. Nespoux et al. (7) reviewed the renoprotective mechanism of sodium-glucose cotransporter 2 (SGLT2) inhibitors, which downregulate tubular reabsorption-induced early glomerular hyperfiltration. Garson et al. (8) revealed that podocytes mediate glomerular transendothelial albumin passage via endothelin-1-regulated heparanase expression. Lai et al. (9) revealed the importance of cell-to-cell communication between different glomerular cell types in DKD using podocyte and endothelial-specific elimination of bone morphogenetic protein and activin membrane-bound inhibitor (BAMBI) expression in streptozotocin-induced diabetic endothelial nitric oxide synthase (eNOS)-deficient and control eNOS-deficient mice. Unfortunately, these studies merely highlight the limited types of cell-to-cell interactions in DKD, and detailed insight into cell communication in DKD is lacking.

Single-cell sequencing (scRNA-seq) is a technological evolution and provides unprecedented insight into cell communication (10–12). In experimental studies of renal diseases (13–16), scRNA-seq technology furthers the understanding of the mechanisms and cell-to-cell interactions involved in disease pathogenesis. In human kidneys (17–19), scRNA-seq has helped to identify novel cell types, reveal potential mechanisms, and investigate cell communication from distinct aspects. Lake et al. (17) primarily deciphered the cell types, distributions, cell differentiation, and cell-to-cell interactions based on integrins in normal human kidneys. A study on lupus nephritis (18) highlighted the immune cells, immune-associated mechanisms, and cell-to-cell interactions based on the functions of chemokines and cytokines. These studies merely described the specific patterns of cell cross-talk. Moreover, cross-talk has not been fully elucidated in DKD (19).

In this study, we provided an overall perspective of cell communication in human DKD based on a single-nucleus transcriptomic dataset. In addition, the relationships between hub genes involved in cell communication and renal function were determined. This study of cell communication between individual cells based on ligand-receptor interactions in DKD may facilitate the discovery of novel mechanisms, biomarkers, and drug targets to better serve patients.

## Materials and Methods

### Single-Nucleus Transcriptomic Data Preparation

First, we downloaded snRNA-seq data from the Gene Expression Omnibus (https://www.ncbi.nlm.nih.gov/geo) dataset GSE131882, which contained the single-nucleus transcriptomic data of three nondiabetic controls and three patients with early DKD produced by 10× Genomics.

### Cell Type Identification

The raw gene expression matrix was obtained and processed to align reads with the reference genome (Homo_sapiens_GRCh38_96) using Cell Ranger (version 4.0.0). Data filtration and normalization were performed using the R package Seurat (version 3.1.1) according to the manufacturer's manual (http://satijalab.org/seurat/) (20). Nuclei with at least 200 genes and percentage of mitochondrial DNA-derived gene expression <25% and genes expressed in at least one single nucleus were retained in the subsequent analysis; otherwise, they were removed. Only snRNA-seq data that met quality control criteria were analyzed in this study.

Further, t-distributed stochastic neighbor embedding (t-SNE) was performed for unsupervised clustering using the R package Seurat (version 3.1.1). Subclustering of specific cell types was performed using OmicStudio (https://www.omicstudio.cn/tool). Annotation of all clusters and subclusters was manually performed based on known cell-type marker genes (17, 18).

### Deferentially Expressed Genes in Specified Clusters

After cell annotation, differentially expressed genes (DEGs) in specified cell-types were analyzed using the FindMarkers function based on the bimod algorithm of the R package Seurat (version 3.1.1). Fold changes ≥1.25 and *p* < 0.05 were considered significantly modulated.

### Cell Cross-Talk Analysis

CellPhoneDB (21) is a public repository of curated receptors, ligands and their interactions. In this study, cell cross-talk interaction was performed using CellPhoneDB (version 2.1.1) according to the manufacturer's manual (https://www.cellphonedb.org/). The mean value represents the average ligand and receptor expression in a specific cell type, which is calculated based on the percentage of cells expressing the specific gene and the gene expression mean. The *P*-value is calculated based on the proportion of the means that are as high as or higher than the actual mean, which represents the likelihood of a specific cell type of a given receptor–ligand complex.

### Protein Expression and Immunochemistry Analysis

The protein expression determined using immunochemistry was obtained from The Human Protein Atlas (https://www.proteinatlas.org/).

### Clinicopathological Correlation Analysis

Nephroseq is a free platform for integrative data mining, including genotype data and phenotype data. The two datasets in Nephroseq, Woroniecka Diabetes Glom, and Woroniecka Diabetes TubInt (22), were analyzed in this study. Pearson's correlation analysis between hub genes and glomerular filtration rate (GFR) in patients with DKD was performed using Nephroseq v5 according to the manufacturer's manual (http://v5.nephroseq.org).

### Statistical Analysis and Data Visualization

Statistical analysis was performed using SPSS 22.0 (SPSS Inc., USA). The figures were illustrated using OmicStudio, GraphPad Prism 7.0 (GraphPad Software Inc., La Jolla, USA), and Microsoft PowerPoint (Microsoft Inc., Redmond, USA).

## Results

### Identifications of Renal Cells and Immune Cells

**Figure 1:**
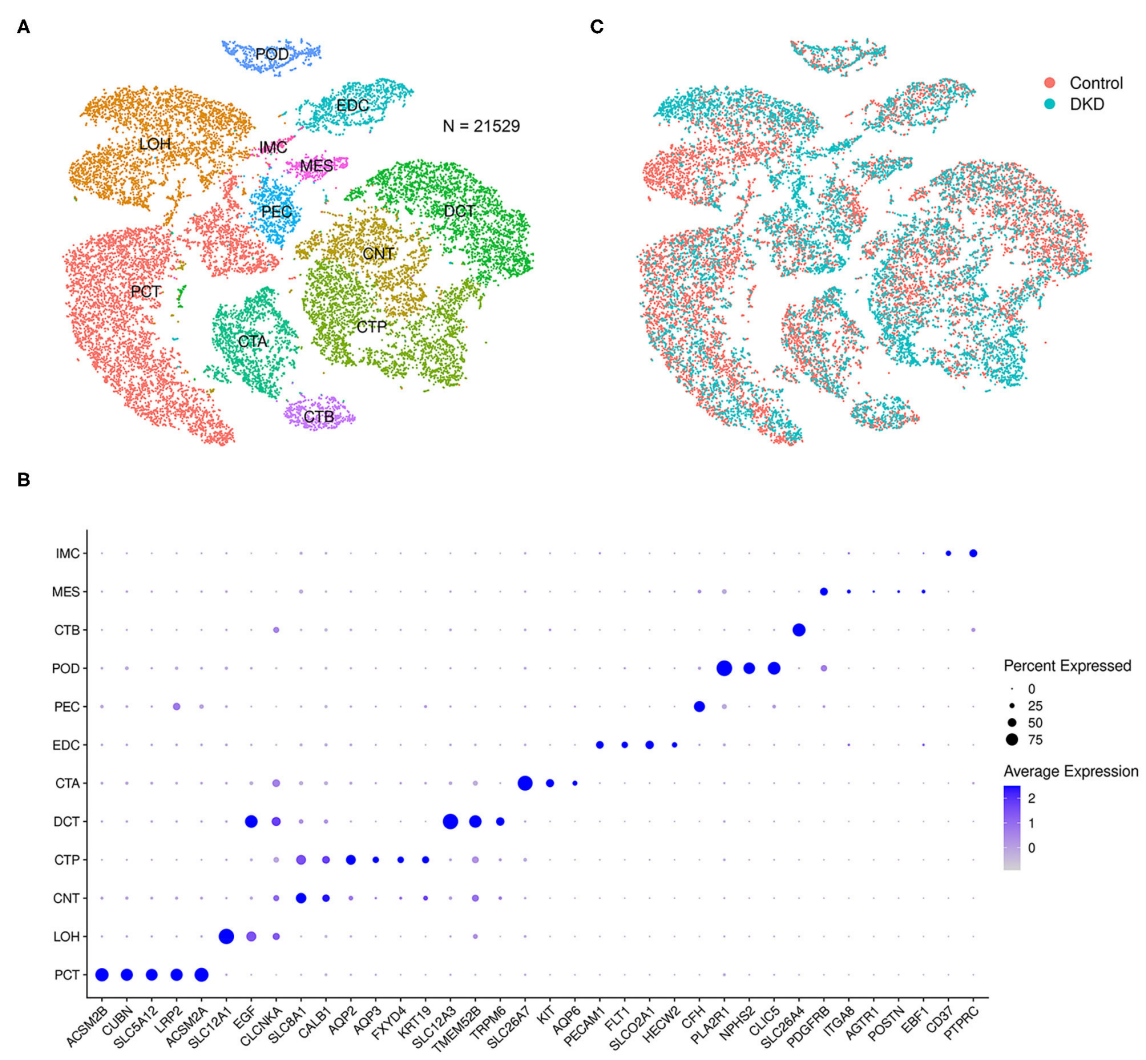
Integrated snRNA-seq of nondiabetic and diabetic kidneys. We performed t-SNE analysis **(A)**, identified gene markers **(B)**, and determined the distributions **(C)** of the 21,529 nuclei. Proximal tubular convoluted cells (PCT), cells of the loop of Henle (LOH), connecting tubule cells (CNT), principal cells of the collecting duct (CTP), distal convoluted duct cells (DCT), intercalated cell A from the collecting duct (CTA), glomerular endothelial cells (EDC), parietal epithelial cells (PEC), podocytes (POD), intercalated cell B from the collecting duct (CTB), mesangial cells (MES), and immune cells (IMC) were manually identified.

After data filtration, the number of nuclei analyzed in the current study was 21,529. According to the known markers, we manually identified proximal tubular convoluted cells, cells of the loop of Henle, connecting tubule cells, principal cells of the collecting duct, distal convoluted duct cells, intercalated cell A from the collecting duct, endothelial cells, parietal epithelial cells, podocytes, intercalated cell B from the collecting duct, mesangial cells, and immune cells (Figure 1A). The markers used in this study and the distributions of disparate cells in the different groups are shown in Figures 1B,C, respectively.

**Figure 2:**
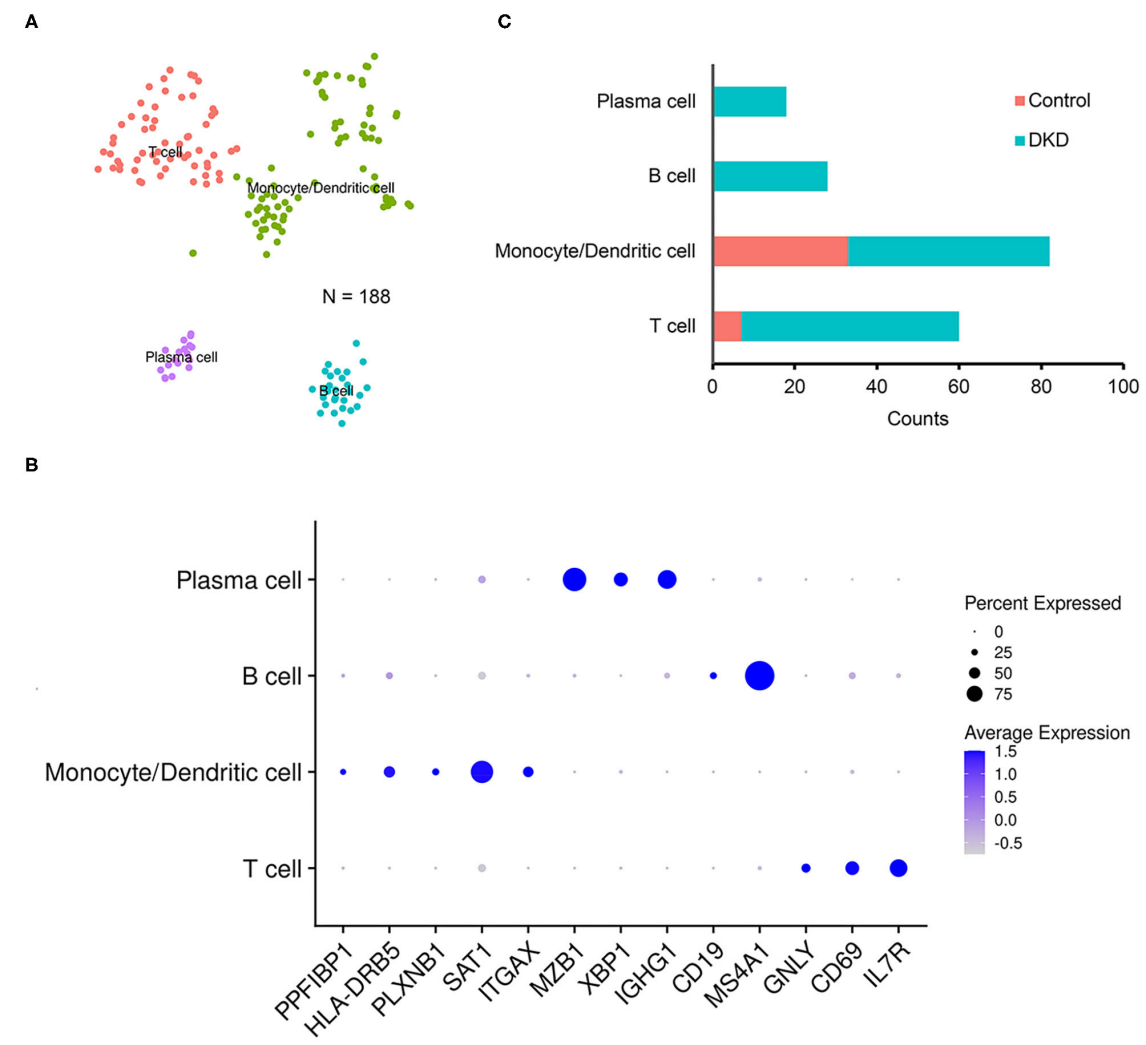
Immune cells in non-diabetic and diabetic kidneys. We performed t-SNE analysis **(A)**, identified gene markers **(B)**, and determined the distributions **(C)** of the subclustered immune cells. T cells, monocytes and dendritic cells, B cells, and plasma cells were manually identified.

Notably, the number of immune cells was significantly increased in the DKD group (DKD vs. control, 148 nuclei vs. 40 nuclei, *p* < 0.05). To determine the types of immune cells, we performed subcluster analysis using t-SNE in immune cells (188 nuclei) and found that renal immune cells comprise T cells, monocytes, dendritic cells, B cells, and plasma cells (Figure 2A) using reported marker genes (Figure 2B). In nondiabetic controls, T cells, monocytes, and dendritic cells consist of renal immune cells. In the DKD group, the total number of immune cells was increased, and numbers of T cells, monocytes, and dendritic cells were increased (Supplementary Table 1). Intriguingly, all B cells and plasma cells newly accumulated in the DKD group (Figure 2C).

### DEGs in Specific Cell Types

Next, we analyzed the DEGs of specific cell types.

**Figure 3:**
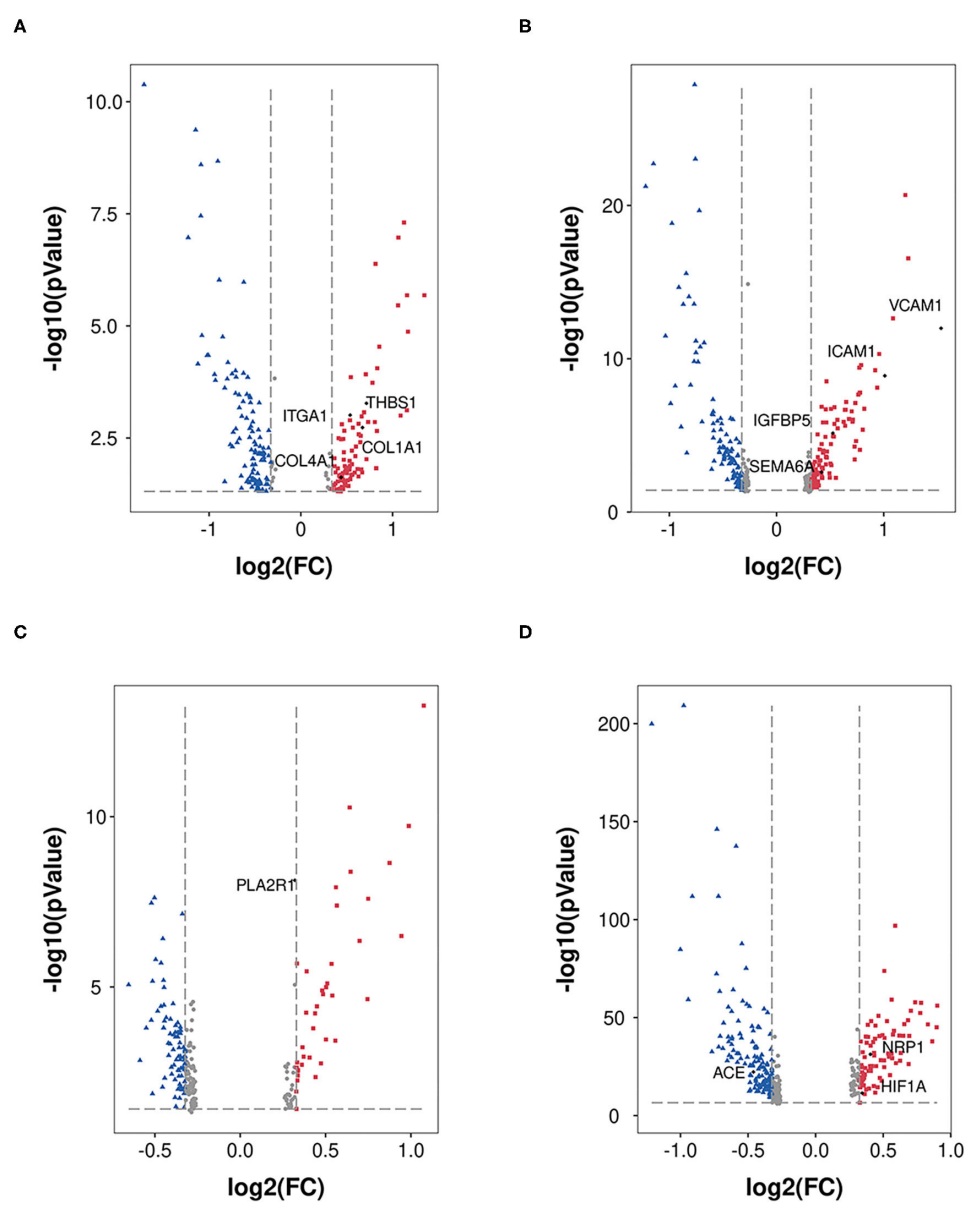
DEGs in MES, EDC, POD, and PCT. DEGs (DKD vs. nondiabetic control) were analyzed in MES **(A)**, EDC **(B)**, POD **(C)**, and PCT **(D)**. The dots in red represent upregulated genes, and the dots in blue represent downregulated genes.

In the mesangial cells (399 nuclei), 88 upregulated genes (the top five genes were SLC2A3, RIPOR3, CCN1, RGS16, and HSPA1A) and 141 downregulated genes (the top five genes were TSC22D3, SPARCL1, ZFAND5, MT1X, and PIK3R1) were identified (Figure 3A). Extracellular environment-related genes (THBS1, ITGA1, COLA1, and COL4A1) are upregulated in DKD.

Figure 3B shows the 102 upregulated genes (the top five genes were VCAM1, SLC2A3, FOS, EMP1, and ICAM1) and 105 downregulated genes (the top five genes were PDK4, TSC22D3, DDIT4, MT1M, and MT-CYB) in EDC (1,079 nuclei). Moreover, the levels of indicators of injury (IGFBP5 and SEMA6A) were increased.

A total of 663 podocytes were analyzed, and we determined that the levels of 39 genes were increased (the top five genes were FOS, EGR1, NR4A1, JUN, and MYADM), while the levels of 80 genes were reduced (the top five genes were GLUL, GPX3, GADD45B, MT-ATP6, and PRMT1; Figure 3C). In addition, PLA2R had no significant alteration according to our analysis.

We analyzed proximal tubular convoluted cells (5,474 nuclei) regarding its crucial roles in reabsorption and glomerulotubular balance and determined 84 upregulated genes (the top five genes were HSPA1A, SOX4, VCAM1, HIST1H2AC, and PROM1) and 134 downregulated genes (the top five genes were FKBP5, FTL, FTCD, TIPARP, CYP3A5; Figure 3D). The expression of HIF1A and NRP1 was increased, and the expression of ACE was decreased. Nevertheless, we did not find a significant change in the expression of ACE2.

### Cell Cross-Talk in DKD

To reveal the cellular communication in the kidney of DKD, we performed an analysis based on receptor-ligand interactions using CellPhoneDB.

**Figure 4:**
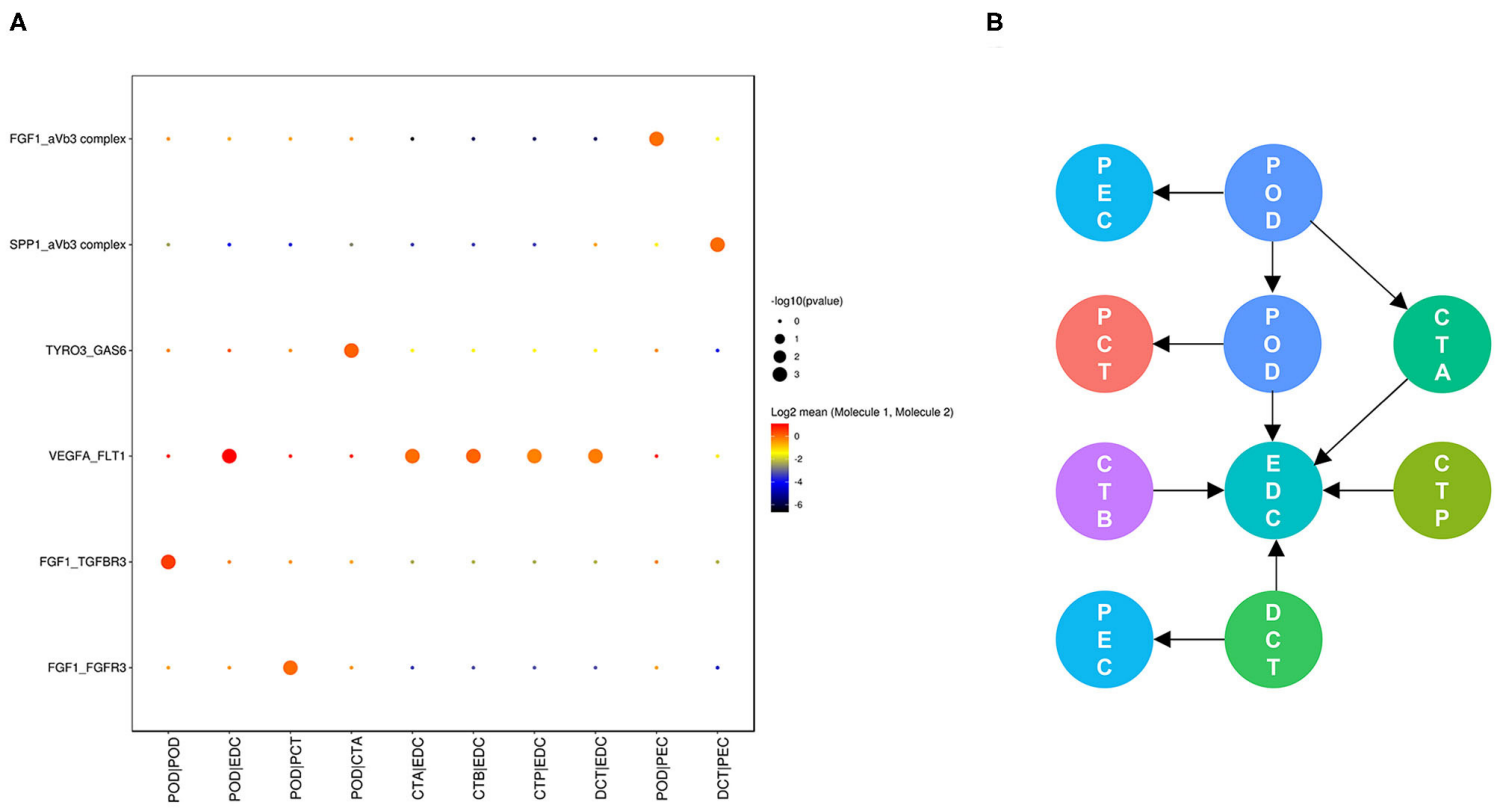
Cell cross-talk in the kidneys of nondiabetic patients. We analyzed all individual cells based on ligand-receptor interactions to reveal the cell cross-talk in the kidneys of nondiabetic patients **(A)**. The involved cell types are summarized and the thickness of the arrow represents the number of interactions **(B)**.

Cell communication in nondiabetic kidneys is defined as basic cell communication that maintains normal renal function (Figure 4). We found that glomerular endothelial cell-expressed FLT1 and podocyte-expressed VEGFA and FGF1 are key molecules (Figure 4A) and that glomerular endothelial cells together with podocytes play crucial roles in glomerular and glomerulotubular cell cross-talk.

**Figure 5:**
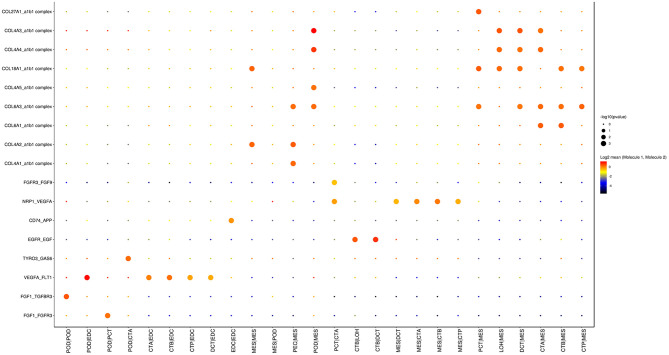
Cell cross-talk in the kidneys of diabetic patients. We analyzed all individual cells based on ligand-receptor interactions to reveal the cell cross-talk in the kidneys of diabetic patients.

**Figure 6:**
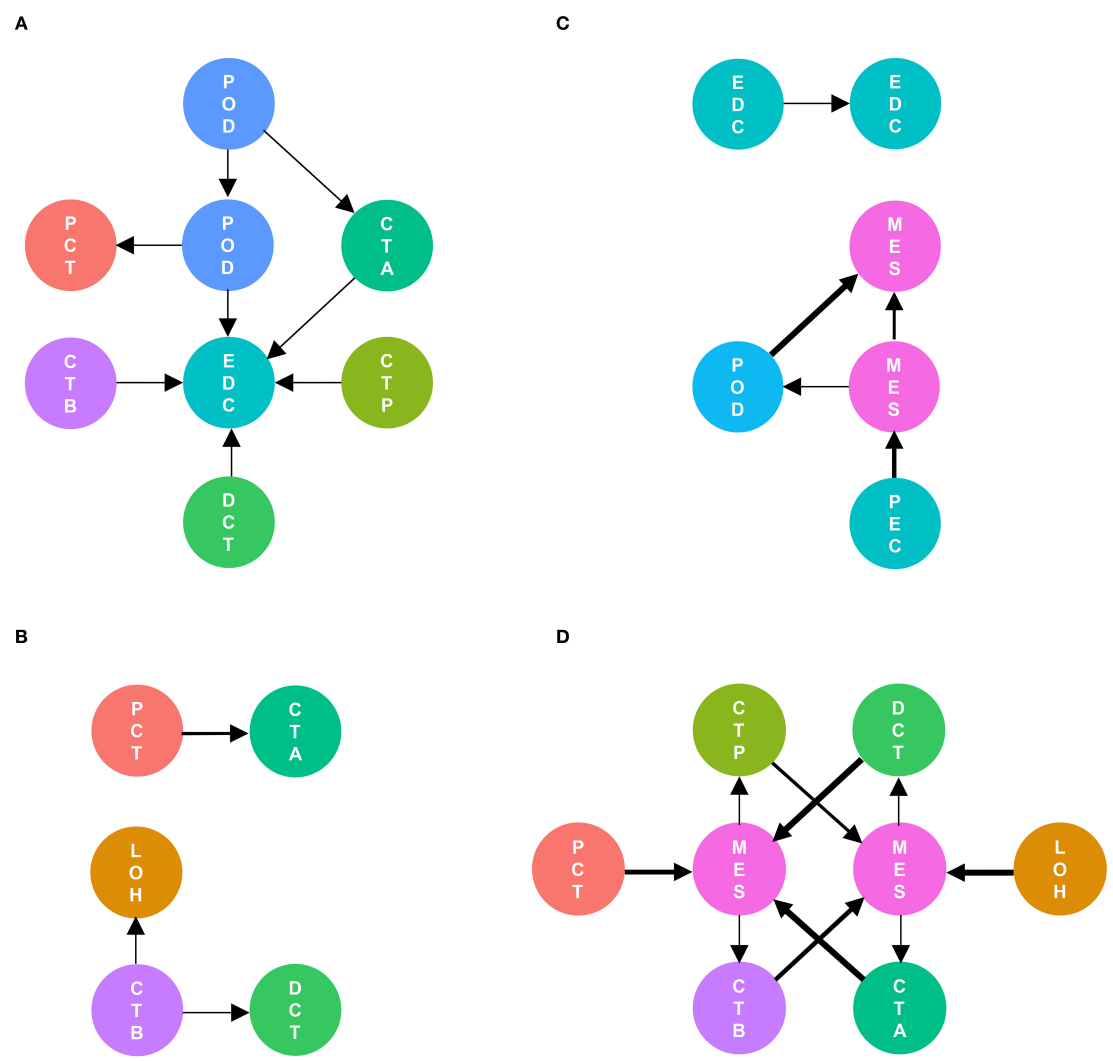
Different types of cells are activated in cell communication in DKD. The involved cell types are summarized and the thickness of the arrow represents the number of interactions. We divided the cell cross-talk into four groups: same cell cross-talk between nondiabetic and diabetic kidneys **(A)**, cell cross-talk in tubules **(B)**, cell cross-talk in glomeruli **(C)**, and glomerulotubular cell cross-talk **(D)**.

As shown in Figure 5, cell communication was significantly altered in DKD conditions. The most noticeable change is the activation of integrin pathways in glomerular and glomerulotubular cell cross-talk. In addition, we noticed that both glomerular and tubular NRP1 participate in the enhanced cell cross-talk of DKD. As shown in Figure 6, we summarized different types of cell communications separately. Figure 6A shows the impairment of basic cell communication and reveals the reduction of the podocyte-expressed FGF1-to-PEC-expressed aVb3 complex and DCT-expressed SPP1-to-PEC-expressed aVb3 complex. Conversely, Figure 6B indicates that the cell cross-talk in the tubule was markedly enhanced. Moreover, we found that mesangial cells were strongly activated in both glomerular (Figure 6C) and glomerulotubular (Figure 6D) cell cross-talk.

### Genes Involved in Cell Cross-Talk Are Associated With Renal Function

**Figure 7:**
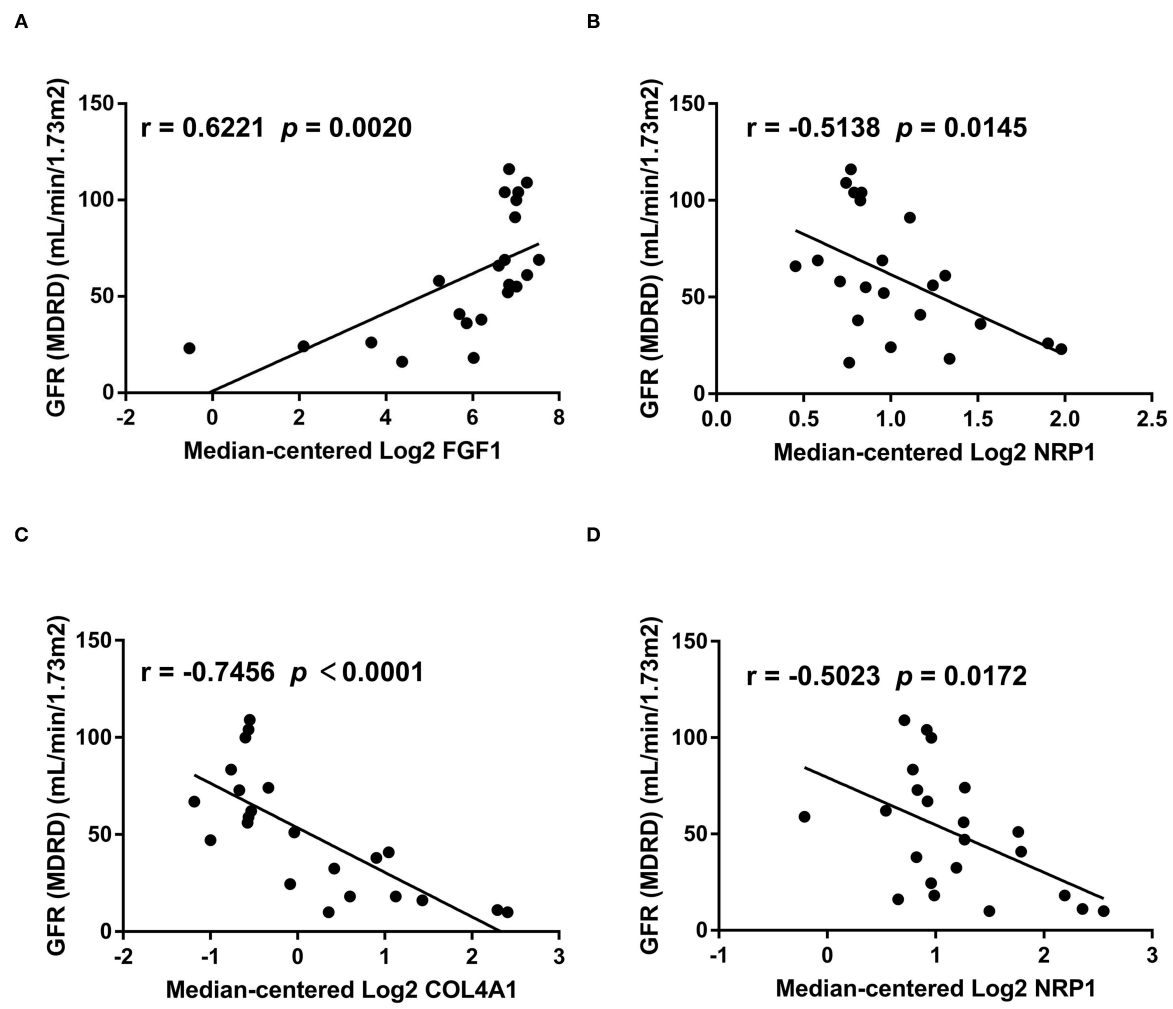
The relationship between hub genes and GFR. We analyzed the relationship between genes involved in cell cross-talk and GFR based on bulk sequencing data. The levels of glomerular FGF1 **(A)** are positively associated with the levels of GFR, while the levels of glomerular NRP1 **(B)**, tubular COL4A1 **(C)**, and tubular NRP1 **(D)** are negatively related to the levels of GFR.

Finally, we investigated the relationship between hub genes involved in cell communication and renal function. Glomerular FGF1 expression (Figure 7A) was positively associated with the levels of GFR, while the levels of glomerular NRP1 (Figure 7B), tubular COL4A1 (Figure 7C), and tubular NRP1 (Figure 7D) were negatively related to the levels of GFR, suggesting that cell cross-talk-related mechanisms contribute to the development of DKD. These findings implied that the hub genes may have potential roles in the diagnosis and prevention of DKD.

## Discussion

Cell cross-talk participates in the development of DKD. Based on a snRNA-seq dataset and two bulk gene datasets, we provided new insight into cell communication and genes involved in DKD.

In 2019, Fu et al. (14) primarily performed scRNA-seq analysis in streptozotocin-induced diabetic endothelial nitric oxide synthase (eNOS)-deficient and control eNOS-deficient mice and revealed increased infiltrating macrophages in glomeruli, dynamic alterations in the pattern of expressed genes in glomerular endothelial cells and mesangial cells of DKD and control mice, and variable responses of individual cells. In addition, this study preliminarily analyzed the cell cross-talk between glomerular individual cells based on ligand-receptor analysis. In the same year, Wilson et al. (19) performed snRNA-seq analysis in DKD for the first time and revealed the significance of increased potassium secretion and angiogenic and possible ligand-receptor signaling pathways in glomerular individual cells. Regretfully, the former studies have limitations. First, only the cell-to-cell interactions between glomerular individual cells were reported. Second, the subunit architecture of ligands and receptors, which accurately represents heteromeric complexes, was not taken into account. This is crucial, as cell cross-talk interacts mediated by multisubunit protein complexes instead of the binary representation used in the previous study (19). Third, the relationship between cell communication-related genes and clinical indicators was not elucidated. In this study, we analyzed cell cross-talk in all individual cells in human kidneys using a novel method (20), which accurately represents heteromeric complexes, and revealed the relationship between hub genes involved in cell cross-talk and renal function. This study reveals further mechanisms and indicates novel biomarkers and potential therapeutic targets.

Cell-to-cell interactions in the same cell type play important roles in both nondiabetic and diabetic kidneys. Podocyte-to-podocyte interactions possibly maintain the basic function of the kidney, which needs to be further studied. In DKD, mesangial cell proliferation contributes to increased internal communication via integrin pathways. Moreover, the internal communication of glomerular endothelial cells via CD74-APP is increased. CD74 (23) is upregulated in diabetic retinopathy with proliferative lesions, and APP (24, 25) is increased in diabetic microvascular complications, indicating a potential mechanism by which the angiogenesis mediated by CD74 and APP participates in DKD development and progression.

Cell-to-cell interactions limited into glomerular or tubular individual cell types are changed in DKD. In glomeruli, podocyte-expressed FGF1-mediated cell cross-talk is decreased, which is consistent with the former report that the protein levels of glomerular FGF1 are decreased in DKD (26). We found that the levels of glomerular FGF1 are positively related to the levels of GFR, suggesting that FGF1 may contribute to the development of DKD. Previous studies (26, 27) showed that FGF1 supplementation ameliorates DKD due to anti-inflammatory and antioxidative stress mechanisms, suggesting that FGF1 is a renoprotetctive factor and an encouraging therapeutic target in DKD. In tubules, PCT-expressed NRP1-mediated cell cross-talk was increased, and NRP1 expression was upregulated (Supplementary Figure 1). We also found that FGF1 expression is negatively associated with GFR, suggesting a potential NRP1-regulated mechanism in DKD. However, a previous study (28) showed a low density of NRP1 expression and downregulated NRP1 levels in renal fibrosis, which is contradictory to our findings. To elucidate the role of NRP1 in DKD, more samples including different disease statuses need to be collected, and further studies are needed.

In the DKD groups, we deciphered active glomerulotubular cell-to-cell interactions. In a tubule-centric view (29), the upregulation of SGLT1 and SGLT2 in PCT induced the alteration of glomerulotubular communication and hyperfiltration, explaining the renoprotective mechanisms of the novel agent SGLT2 inhibitor in DKD treatment (30–32). Nevertheless, the levels of SGLT1 and SGLT2 were not significantly altered in PCT in this study. Individual differences and different disease statuses may lead to contradictory results.

Finally, cell identification revealed immune cells in kidneys. Interestingly, DKD with high IFTA (interstitial fibrosis and tubular atrophy) samples contributed all identified B cells, suggesting the crucial role of B cells in DKD (Supplementary Figures 2A,B). We performed DEG analysis in immune cells and found that CD20 expression was significantly increased in the DKD groups (Supplementary Figure 2C). Some studies have revealed that targeting CD20 achieves therapeutic effects in renal diseases. An experimental study showed the protective effects of CD20 antibodies in lupus mice (33). Clinical evidence provides that CD20 antibodies achieve therapeutic effects in recurrent focal segmental glomerulosclerosis (34) and membranous nephropathy (35). However, the role of B cells in DKD has not been fully elucidated (36). Increased IgG+ B cells were found in the glomeruli of diabetic NOD mice when compared with those in nondiabetic mice, suggesting that B cells may contribute to the pathogenesis and prognosis of DKD (37). In DM patients, Zhang et al. (38) found increased CD38+CD19+ B cell counts in the peripheral blood. Moreover, the number of CD38+CD19+ B cells was positively correlated with the 24 h urinary protein concentration and was reduced after treatment. Taken together, these findings suggest that B cells may participate in the development of DKD. We speculate that agents targeting B cells or CD20 antibodies may have promising therapeutic effects in DKD, which needs to be further studied in future research.

Regretfully, snRNA-seq has its own limitation in capturing immune cell populations due to nanodrop technology, and frozen or optimal cutting temperature compounds may lead to the loss of information. Thus, the cross-talk between immune cells and renal parenchymal cells in DKD was not fully deciphered in this study. In addition, larger sample sizes and conditional knockout models are needed to better elucidate cell cross-talk and its further mechanism in DKD.

## Conclusion

In summary, this study revealed cell cross-talk based on snRNA-seq and the associations between genes involved in cell communication and renal function in DKD. In DKD, cell-to-cell interactions via integrin pathways are increased, mesangial cells are stimulated and glomerulotubular communication is strongly enhanced. The level of glomerular FGF1 is positively associated with the level of GFR, while the levels of glomerular NRP1, tubular COL4A1, and tubular NRP1 are negatively associated with the level of GFR. This study furthers our understanding of cell cross-talk in DKD and reveals novel mechanisms, new biomarkers, and potential therapeutic targets to benefit patients.

## Data Availability Statement

The datasets presented in this study can be found in online repositories. The names of the repository/repositories and accession number(s) can be found in the article/Supplementary Material.

## Author Contributions

YW designed the study, performed the data analysis, and wrote the first draft. XG and ZJ revised the draft. AL and ML helped improve the methodology. All authors contributed to the article and approved the submitted version.

## Conflict of Interest

The authors declare that the research was conducted in the absence of any commercial or financial relationships that could be construed as a potential conflict of interest.

## Supplementary Material

Supplementary Figure 1 The expression of NRP1 in tubules. The protein levels of NRP1 in normal human kidneys were obtained from The Human Protein Atlas **(A)**. Tubular NRP1 is upregulated in DKD based on bulk sequencing data **(B)**. ^***^*p* < 0.001.

Supplementary Figure 2 The distribution and DEGs of IMC. The distribution of IMCs according to disease **(A)** and IFTA **(B)** is presented, and DEGs (DKD vs. nondiabetic control) in IMCs were calculated **(C)**. The dots in red represent upregulated genes, and the dots in blue represent downregulated genes.

Supplementary Table 1 Number of immune cells in each sample.
