# Supplementary figures and images for: Single-Nucleus Transcriptomic Analysis Reveals Important Cell Cross-Talk in Diabetic Kidney Disease
Source: Front Med (Lausanne). 2021 Apr 21;8:657956. doi: 10.3389/fmed.2021.657956 (PMC8097156; doi:10.3389/fmed.2021.657956)

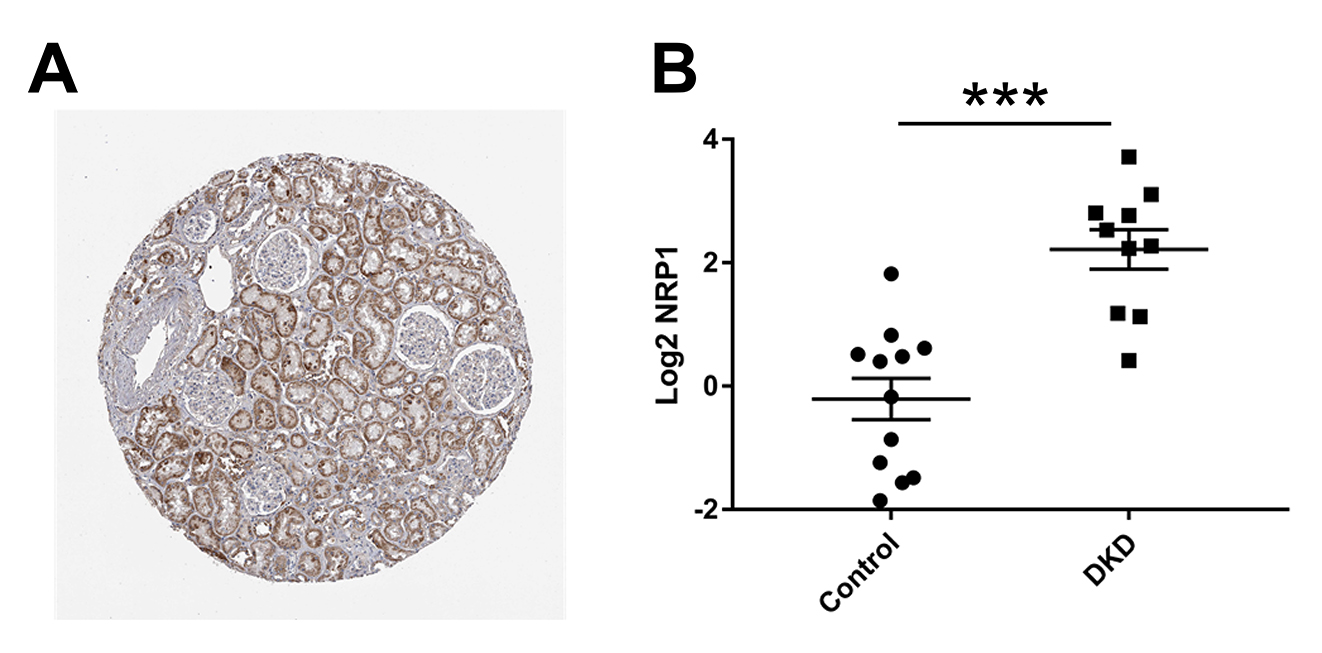

Supplement: Supplementary Figure 1 — The expression of NRP1 in tubules. The protein levels of NRP1 in normal human kidneys were obtained from The Human Protein Atlas (A). Tubular NRP1 is upregulated in DKD based on bulk sequencing data (B). ***p < 0.001. [file Image_1.JPEG]

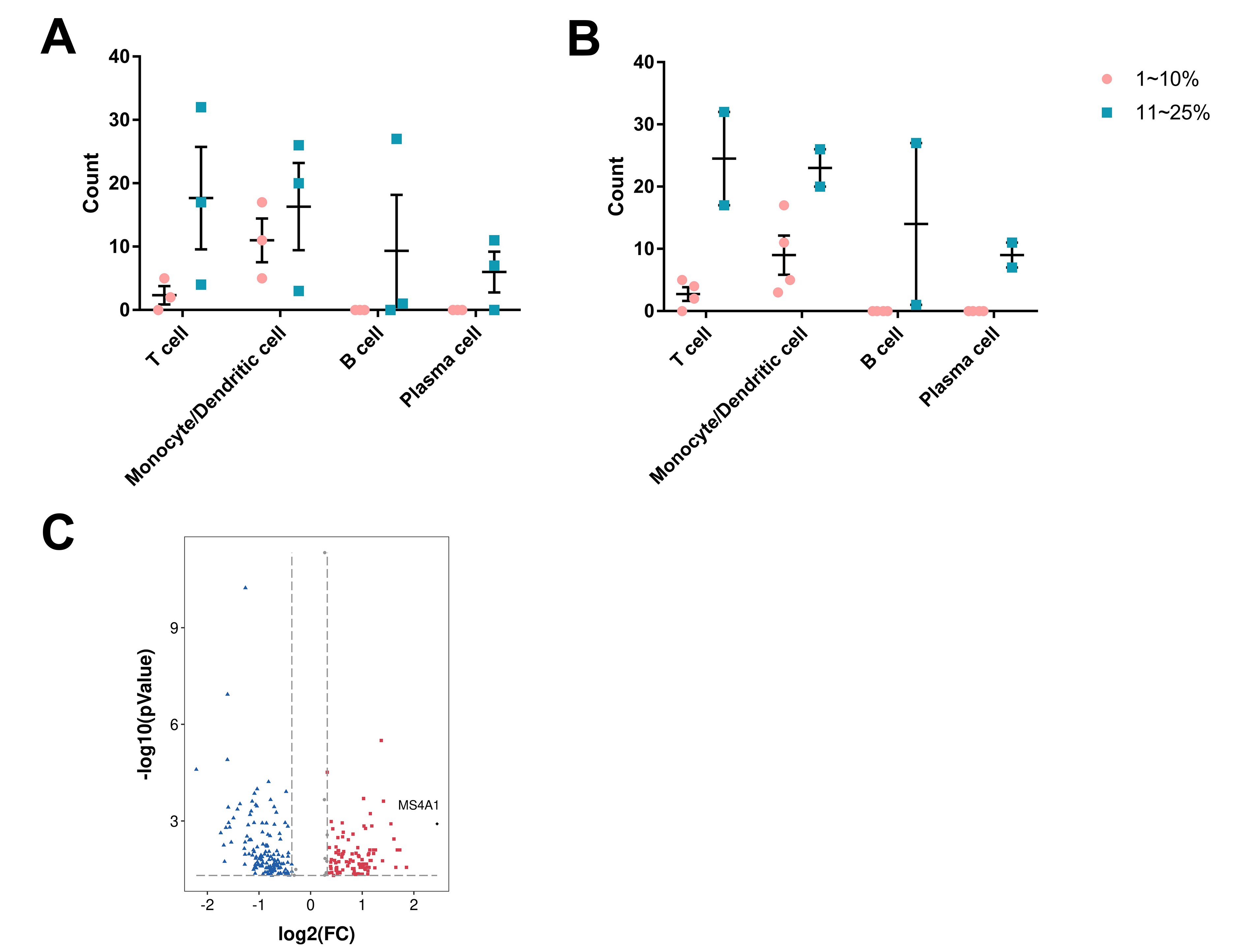

Supplement: Supplementary Figure 2 — The distribution and DEGs of IMC. The distribution of IMCs according to disease (A) and IFTA (B) is presented, and DEGs (DKD vs. nondiabetic control) in IMCs were calculated (C). The dots in red represent upregulated genes, and the dots in blue represent downregulated genes. [file Image_2.JPEG]
