# Supplementary material for: Single-Nucleus Transcriptomic Analysis Reveals Important Cell Cross-Talk in Diabetic Kidney Disease
Source: Front Med (Lausanne). 2021 Apr 21;8:657956. doi: 10.3389/fmed.2021.657956 (PMC8097156; doi:10.3389/fmed.2021.657956)
Supplement: Supplementary Table 1 — Number of immune cells in each sample. [file Table_1.DOCX]

**Table S1. Number of immune cells in each sample.**

| Count | T cell | Monocyte/  Dendritic cell | B cell | Plasma cell |
| --- | --- | --- | --- | --- |
| gsm3823939_c1 | 5 | 17 | 0 | 0 |
| gsm3823940_c2 | 0 | 11 | 0 | 0 |
| gsm3823941_c3 | 2 | 5 | 0 | 0 |
| gsm3823942_d1 | 32 | 26 | 1 | 7 |
| gsm3823943_d2 | 17 | 20 | 27 | 11 |
| gsm3823944_d3 | 4 | 3 | 0 | 0 |
